# Electric Field–Controlled Multistep Proton Evolution in H*_x_*SrCoO_2.5_ with Formation of H–H Dimer

**DOI:** 10.1002/advs.201901432

**Published:** 2019-08-15

**Authors:** Hao‐Bo Li, Feng Lou, Yujia Wang, Yang Zhang, Qinghua Zhang, Dong Wu, Zhuolu Li, Meng Wang, Tongtong Huang, Yingjie Lyu, Jingwen Guo, Tianzhe Chen, Yang Wu, Elke Arenholz, Nianpeng Lu, Nanlin Wang, Qing He, Lin Gu, Jing Zhu, Ce‐Wen Nan, Xiaoyan Zhong, Hongjun Xiang, Pu Yu

**Affiliations:** ^1^ State Key Laboratory of Low Dimensional Quantum Physics and Department of Physics Tsinghua University Beijing 100084 China; ^2^ Key Laboratory of Computational Physical Sciences (Ministry of Education) State Key Laboratory of Surface Physics, and Department of Physics Fudan University Shanghai 200433 China; ^3^ National Center for Electron Microscopy in Beijing Key Laboratory of Advanced Materials (MOE) Beijing 100084 China; ^4^ State Key Laboratory of New Ceramics and Fine Processing School of Materials Science and Engineering Tsinghua University Beijing 100084 China; ^5^ Beijing National Laboratory for Condensed Matter Physics Institute of Physics Chinese Academy of Sciences Beijing 100190 China; ^6^ Collaborative Innovation Center of Quantum Matter Beijing 100084 China; ^7^ International Center for Quantum Materials School of Physics Peking University Beijing 100871 China; ^8^ Department of Mechanical Engineering Tsinghua University Beijing 100084 China; ^9^ Advanced Light Source Lawrence Berkeley National Laboratory Berkeley 94720 CA USA; ^10^ Department of Physics Durham University Durham DH13LE UK; ^11^ School of Physical Sciences University of Chinese Academy of Sciences Beijing 100049 China; ^12^ Collaborative Innovation Center of Advanced Microstructures Nanjing 210093 China; ^13^ RIKEN Center for Emergent Matter Science (CEMS) Saitama 351‐0198 Japan

**Keywords:** brownmillerite, charge‐neutral H–H, ionic liquid gating, protonation

## Abstract

Ionic evolution–induced phase transformation can lead to wide ranges of novel material functionalities with promising applications. Here, using the gating voltage during ionic liquid gating as a tuning knob, the brownmillerite SrCoO_2.5_ is transformed into a series of protonated H*_x_*SrCoO_2.5_ phases with distinct hydrogen contents. The unexpected electron to charge‐neutral doping crossover along with the increase of proton concentration from *x* = 1 to 2 suggests the formation of exotic charge neutral H–H dimers for higher proton concentration, which is directly visualized at the vacant tetrahedron by scanning transmission electron microscopy and then further supported by first principles calculations. Although the H–H dimers cause no change of the valency of Co^2+^ ions, they result in clear enhancement of electronic bandgap and suppression of magnetization through lattice expansion. These results not only reveal a hydrogen chemical state beyond anion and cation within the complex oxides, but also suggest an effective pathway to design functional materials through tunable ionic evolution.

## Graphical abstract

**An ionic liquid gating strategy** is proposed, and then a series of H*_x_*SrCoO_2.5_ phases with distinct hydrogen contents is discovered. The unexpected electron to charge‐neutral doping crossover suggests the formation of exotic H–H dimers for higher hydrogen concentration, which is directly visualized at the vacant tetrahedron of the lattice by scanning transmission electron microscopy and supported by theoretical calculations.

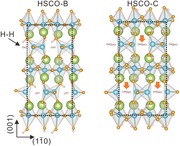

## 1 Introduction

Ionic evolution–induced material engineering forms a productive avenue for designing novel material functionalities with great potential applications in various aspects.^1^, ^2^, ^3^, ^4^, ^5^, ^6^, ^7^, ^8^ Especially, the hydrogen ion attracts particular attention due to its comparatively small radius as well as easy accessibility. Several pathways have been proposed to achieve hydrogen ion exchange and evolution, such as thermal annealing with metal hydride,^9^, ^10^, ^11^, ^12^ hydrogen incorporation with catalytic noble metals,^13^, ^14^, ^15^ and ionic liquid gating (ILG)‐induced protonation.^4^, ^16^ For the former case, the employed metal hydride agent (e.g., NaH and CaH_2_) can generate a series of reduced perovskite compounds (e.g., CaFeO_2_, LaNiO_2_, SrFeO_2_, etc.)^17^, ^18^, ^19^ as well as transition metal oxyhydrides (e.g., LaSrCoO_3_H_0.7_, BaTiO_3−_ *_x_*H*_x_* SrVO_2_H, SrCrO_2_H, etc.)^9^, ^10^, ^11^, ^12^ via ionic exchange between hydrogen anion (H^−^) and O^2−^. While for the latter two methods, the hydrogen evolution usually introduces the hydrogen cation (proton, H^+^) into the materials systems (e.g., VO_2_, SrCoO_2.5_, WO_3_, etc.)^4^, ^13^, ^14^, ^15^, ^16^, ^17^, ^18^, ^19^, ^20^, ^21^ and leads to the reduction of transition metal ion. Along with these successful investigations, several fundamental and interesting questions emerge. What is the fundamental driving force for the hydrogen ion evolution, and can we obtain a subtle manipulation of this? Can we obtain direct structural evidence of the hydrogen ion occupation? Can the hydrogen ions demonstrate other format in oxide besides the known proton (H^+^) and hydrogen anion (H^−^)?

In this work, we propose a novel strategy to manipulate the proton evolution through the application of external electric voltage across the sample. With this guideline, we achieved a systematic control of the proton evolution within brownmillerite SrCoO_2.5_, where two more hitherto‐unexplored phases H*_x_*SrCoO_2.5_ (*x* ≈ 1.5 and 2.0) are discovered. Furthermore, the identical Co^2+^ valency among these three H*_x_*SrCoO_2.5_ phases clearly indicate the existence of charge neutral hydrogens within the two newly discovered compounds, which are experimentally and theoretically confirmed as H–H dimers located in the intrinsic oxygen vacancy channels in the brownmillerite structure. These discoveries not only provide a novel and practical strategy to control the phase transformations induced by ionic evolution, but also unveil an intriguing hydrogen chemical state in protonated oxides. Furthermore, the structural transformation associated with the formation of H–H dimers opens up a new avenue to manipulate material functionalities with strain engineering due to the large associated chemical expansion.

## 2 Results and Discussion

**Figure 1:**
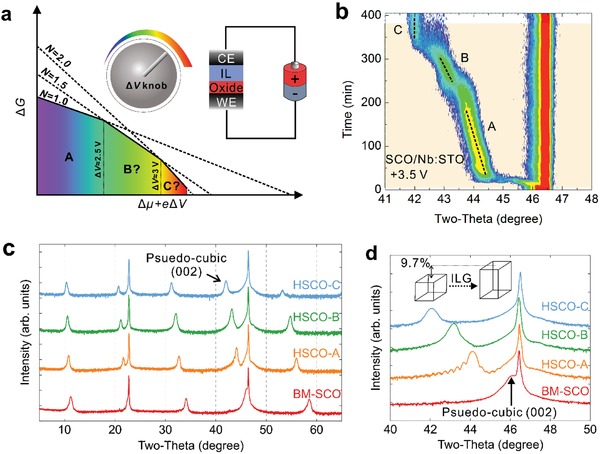
Electric field–controlled multistep phase transitions. a) Schematic illustration of multistep phase transformation driven by electric field tunable chemical potential difference. The working electrode (WE) beneath the oxide and the counter electrode (CE) can build up an electric field across the oxide layer, which acts as a tuning knob to control the chemical potential difference, and eventually forms a series of unexplored phases (B and C) with controllable dopant atomic ratio (***N***). b) In situ XRD scan using chemical potential difference controlled setup. c) Detailed ex situ XRD scans for all three protonated SrCoO_2.5_ phases. The consistency between the ex situ and in situ data suggests that all phases are nonvolatile, and the presence of superlattice peaks indicates that the basic brownmillerite structures remain intact during the ILG. d) The zoom‐in XRD pattern in two‐theta ranging from 40° to 50°. The shift of pseudocubic diffraction peak toward lower angle along the phase transition indicates the large lattice expansion along *c*‐axis.

While different methods might involve distinct mechanisms, the insertion of ions into the material is likely dominated by the chemical potential difference between the associated ions within and outside the materials. During ionic liquid gating, the ILG‐induced electronic double layer (EDL) introduces a nonequilibrium high concentrated proton accumulation at the sample surface (Figure S1a, Supporting Information), which will in turn increase the corresponding chemical potential of protons. When the chemical potential is higher than the corresponding chemical potential inside the material, the proton will be inserted toward the bulk material and eventually achieving a thermal equilibrium state with specific proton concentration within the material. In the convention ILG method (Figure S1b, Supporting Information), this process is driven by the free ionic diffusion of the H^+^, owing to the EDL‐introduced nonequilibrium ion concentrations between the ionic liquid and the thin film (Figure S1b, Supporting Information). Here, we propose a novel strategy to further manipulate the proton evolution through the application of an external electrical voltage across the sample (Figure S1c, Supporting Information). In this case, the bottom electrode (BE) can not only create EDL at thin film surface, but also provide additional electric field over the thin film to tune its corresponding chemical potential difference of the proton, which eventually controls the final state of the protonated SrCoO_2.5_ thin film. To be more specific, as shown in the main text **Figure** **1**a, the change of Gibbs free energy (ΔG) after the hydrogen injection can be fundamentally modified according to $\Delta G\propto -\sum_{i}(\Delta \mu_{i}+\;e\Delta V)N_{i}$, where *N*_i_ indicates the atomic ratio of the element i. Hence in our case, the proton insertion process would be further boosted, and consequently a series of unexplored structural phases with different formation energies and increased hydrogen atomic ratios would emerge when Δ*V* reaches the threshold value for the corresponding phase transformations (Figure 1a).

To testify the proposed voltage‐assisted ILG method, model system SrCoO_2.5_ thin films were grown on both insulating SrTiO_3_ and conducting Nb:SrTiO_3_ (0.5%) (001) substrates, and then characterized with in situ X‐ray diffraction (XRD) with experimental setups shown in Figure S1a,b in the Supporting Information. As shown in Figure 1b, with gating voltage of +3.5 V on the conducting substrate, three subsequent phase transformations were observed in the SrCoO_2.5_/Nb:SrTiO_3_ system, including two new phases with the pseudocubic (002) diffraction peak at ≈43.2° (HSCO‐B) and ≈42.0° (HSCO‐C). This is fundamentally different from the previously reported one‐phase transition from SrCoO_2.5_ to H*_x_*SrCoO_2.5_ (HSCO‐A)^4^ and importantly with the application of negative bias, the phase transition is reversible and the diffraction peak can eventually return to the original position (Figure S2, Supporting Information). It is worth noting that all these three phases can be selectively controlled using different gating voltages (Figure S3, Supporting Information). Therefore, our results strongly suggest that the gating voltage forms an effective parameter to trigger a series of yet unexplored phase transformations. It is important to emphasize that all observed new phases are nonvolatile even with the ionic liquid removed, as confirmed by the ex situ XRD results (Figure 1c), which also reveals that all H*_x_*SrCoO*_y_* phases (HSCO) exhibit the superlattice structure similar to pristine brownmillerite SrCoO_2.5_ (BM‐SCO). As the in‐plane lattice constant is strongly locked to that of the substrate through the phase transformations (Figure S4, Supporting Information), by calculating the (002) pseudocubic peak shift we can conclude that the electric field–induced proton insertion leads to a giant (up to ≈9.7%) chemical expansion along the out‐of‐plane (OOP) film direction (Figure 1d), while the basic brownmillerite framework remains unaffected.

**Figure 2:**
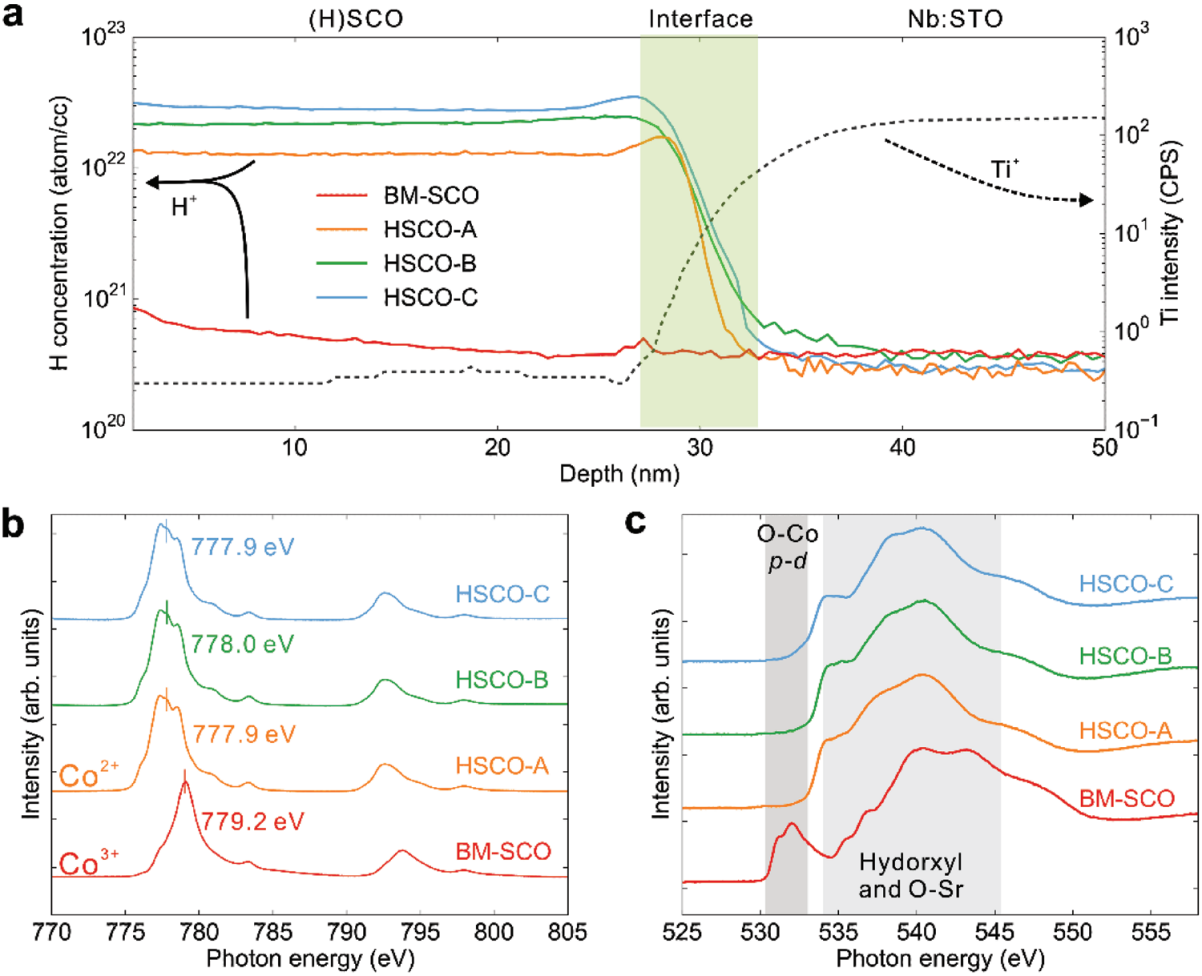
Evidence of the formation of charge neutral hydrogen with the protonated phases. a) Time‐of‐flight secondary‐ion mass spectrometry (TOF‐SIMS) depth profiles of H^+^ and Ti^+^ for all three HSCO phases as well as the reference BM‐SCO phase. The Ti^+^ signal from the Nb:SrTiO_3_ substrate is used as layer marker to identify the interface position between the thin film and the substrate. Soft X‐ray absorption spectra of b) Co *L*‐edge and c) O *K*‐edge for all there protonated phases and brownmillerite phase.

The ILG‐induced oxygen vacancy formation^22^ was carefully ruled out by detailed energy‐dispersive X‐ray spectroscopy (EDS) and X‐ray photoelectron spectroscopy (XPS) measurements (Note S1 and Figure S5a–c, Supporting Information), which singles out hydrogen ion evolution as the dominant factor for the observed phase transformations. The time‐of‐flight secondary‐ion mass spectrometry (TOF‐SIMS) results (**Figure** **2**a) reveal a uniform hydrogen distribution throughout the entire film thickness, with the hydrogen concentrations quantitatively estimated as 0.9, 1.6, and 2.2 H per chemical formula of SrCoO_2.5_ for HSCO‐A, HSCO‐B, and HSCO‐C, respectively. Although, this value could be slightly off from the actual sample composition due to the different matrix elements between the sample (HSCO, SCO) and the standard silicon reference, they provide qualitatively the evolution of hydrogen contents through the phase transformation (Note S1, Supporting Information). Indeed, such a systematic increase of the hydrogen concentration is further supported by the hydrogen forward scattering spectra (HFS) as shown in Figure S5d in the Supporting Information, and both results are consistent nicely with our intuitive expectation as revealed in Figure 1a.

We note that the H^+^ ion (or proton) carries positive charge, and thus can lead to electron doping into the materials,^4^, ^8^ and for instance, the insertion of one proton per Co ion into SrCoO_2.5_ leads to the reduction of Co valence state from 3+ to 2+. Therefore, one might expect a further reduced valence state of Co ions when the H/Co ratio exceeds 1 as in HSCO‐B and HSCO‐C. Surprisingly, the soft X‐ray absorption spectroscopy (sXAS) (Figure 2b) reveals that all three phases (HSCO‐A, B, C) exhibit almost identical features for Co *L*‐edge with characteristic peak at 777.9 ± 0.1 eV as compared to at 779.2 ± 0.1 eV for the reference BM‐SCO sample, which clearly indicates the fact that all three HSCO phases possess identical valence state of Co^2+^.^23^ Therefore, these results strongly suggest the presence of charge neutral hydrogen within the HSCO‐B and HSCO‐C phases, which has not been observed before in complex oxides. Furthermore, the O *K*‐edge results (Figure 2c) show similar suppressed p–d hybridization feature between O and Co ions for all three protonated phases. Both features are also confirmed by the X‐ray photoelectron spectroscopy, as shown in Figure S5b,c in the Supporting Information, and the XPS full scan in Figure S5e in the Supporting Information indicates no other elements are introduced into the materials.

**Figure 3:**
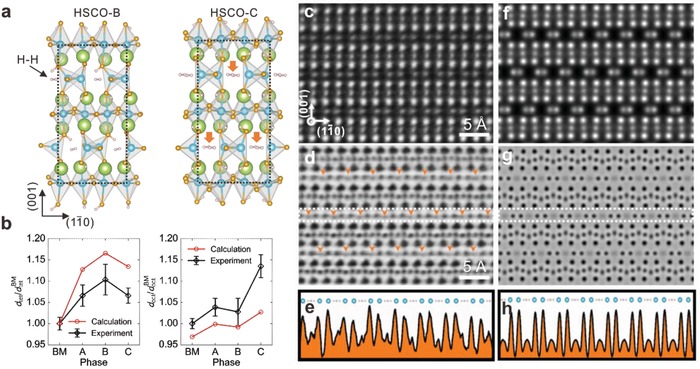
Direct observation of charge neutral H–H dimers. a) Calculated crystalline structures for HSCO‐B and HSCO‐C with formation of H–H dimers. b) Comparison of the experimental and calculated sublayer lattice constants of tetrahedral and octahedral polyhedrons. c) HAADF image, d) ABF image, and e) the line profile of ABF image obtained from the HSCO‐C phase. The zone axis is along pseudocubic [110] direction. The orange arrows indicate the unique structural features contributed by the H–H dimers. The corresponding simulated f) HAADF image, g) ABF image, and h) line profile for ABF image based on calculated HSCO‐C structure.

To gain further structural insights into the atomic structure of the protonated phases, we have performed aberration‐corrected scanning transmission electron microscopy (STEM) studies combined with first‐principles calculations to determine the possible crystalline structures for the two new phases (Notes S2 and S3, Supporting Information). The high‐angle angular dark‐field (HAADF) STEM images (Figure S6a–d, Supporting Information) show clearly alternating stacking of octahedral CoO_6_ and tetrahedral CoO_4_ sublayers as well as the Co–Co dimerization in the tetrahedral layers for BM‐SCO and all HSCO phases, indicating the robustness of the brownmillerite crystalline framework even for substantial hydrogen ion intercalation. The calculations reveal that in the HSCO‐A case, the hydrogen ions bond directly with the interlayer oxygen ion sandwiched between tetrahedral and octahedral sublayers (Figure S6e,f, Supporting Information). When additional hydrogen ions are inserted to form HSCO‐B and HSCO‐C phases, surprisingly the H–H dimers naturally form in the original ordered oxygen channels to minimize the total energy of the entire system (**Figure** **3**a; Figure S6g,h and Figures S7 and S8, Supporting Information). The simulated crystalline structures are nicely in agreement with the experimental results obtained from STEM in both octahedral and tetrahedral sublayers (Figure 3b). Particularly, during the phase transformation from HSCO‐B to HSCO‐C, the orientation of the O—H bond rotates from the tetrahedral sublayer to the octahedral sublayer (Figure S6h, Supporting Information), which may result in the unique transition of the interplanar distance for both *d*_tet_ and *d*_oct_ as shown in Figure 3b.

To acquire direct insights of the charge‐neutral hydrogen, we focus on the HSCO‐C phase for further investigations. While the HAADF‐STEM images (Figure 3c) exhibit mainly the framework of the brownmillerite phase with the observation of mainly Sr and Co ions, the corresponding annular bright field (ABF) images (Figure 3d) capture the signal induced by lighter atoms such as oxygen and hydrogen.^24^ In the HSCO‐C, a unique feature labeled by the arrows in the tetrahedral CoO_4_ layer is observed in both ABF images and the corresponding intensity line profiles (Figure 3e), which well accords with the simulated H–H dimer based on calculated structure in term of both location and contrast (Figure 3f–h). On the other hand, such feature is absent in the HSCO‐A (Figure S9, Supporting Information), which verifies that the feature is unique in HSCO phases with higher hydrogen concentrations. Besides, the existence of O—H groups and charge‐neutral dimers are also verified via room‐temperature infrared spectra (Note S4 and Figures S10 and S11, Supporting Information).

**Figure 4:**
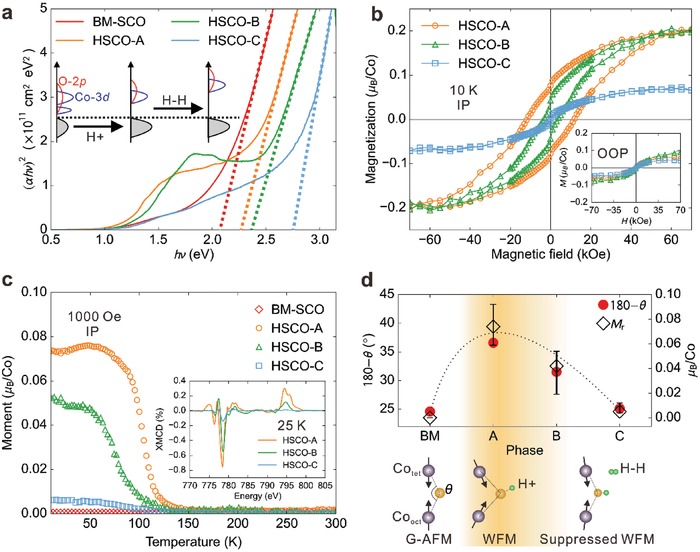
Evolution of the electronic and magnetic states with the formation of H–H dimers. a) Tauc plots ((*αhν*)^2^ vs *hν* plot) for BM‐SCO and other three HSCO phases. The direct optical bandgaps (*E*_g_) are obtained from the fittings of (*αhν*)^2^ = *A*(*hν* − *E*_g_), where *A* is a prefactor. b) In‐plane magnetic hysteresis loops (*M–H* curves) of three HSCO phases measured at 10 K. Inset shows the out‐of‐plane (OOP) magnetization results at the same temperature. c) In‐plane *M–T* curves for all HSCO phases as well as BM‐SCO measured with the magnetic field of 1000 Oe. The inset shows the soft X‐ray magnetic circular dichroism (XMCD) spectra of Co *L*‐edge measured at 25 K with a magnetic field of 2 T applied along the incident direction (30° from the surface to the normal) of the light. d) Summary of experimental in‐plane remnant magnetic moments (*M*_r_) and theoretical calculated bond angles among BM‐SCO and HSCO phases. The dotted line serves as a guideline for the magnetic evolution along with the phase transformation, and the lower panel illustrates the proposed mechanism with the correlation between the canted magnetization and lattice distortion.

Since the formation of H–H dimers leads to merely the structural expansion with robust Co valency, the current system provides an ideal platform to investigate the influence of chemical expansion on the electronic and magnetic states. We note that the large lattice expansion would be corresponding to a large negative pressure, which is usually unachievable with the current high‐pressure approaches. **Figure** **4**a shows the Tauc plots of optical transmittance data obtained at different phases, where a clear blue‐shift of the optical bandgap can be observed, with the direct optical bandgap enhanced from ≈2.08 eV in BM‐SCO to ≈2.28, ≈2.36, and ≈2.75 eV for HSCO‐A, HSCO‐B, and HSCO‐C, respectively. We have also obtained the indirect bandgap from the optical spectra, which increases from ≈0.48 to ≈0.70 eV in the same trend (Figure S12, Supporting Information). Such systematic increase can be attributed to the ascension of both the unoccupied Co‐3d as well as O‐2p states due to the chemical expansion, as suggested by the calculated density of states (Figure S12, Supporting Information).

One of the most interesting results for our previous research in HSCO is the emergence of magnetization in HSCO with proton evolution from the antiferromagnetic SrCoO_2.5_^4^. Interestingly, the magnetic properties of the HSCO phases are also strongly correlated with the hydrogen concentration. As shown in Figure 4b, weak magnetization with well‐defined hysteresis loop was observed for all three HSCO phases. Together with the decreased coercive fields, the remnant magnetization is strongly suppressed from HSCO‐A to HSCO‐B and becomes almost negligible for HSCO‐C. This can also be seen in the temperature‐dependent magnetization measurements (Figure 4c), which reveal that both the spontaneous magnetization as well as its transition temperature are strongly suppressed with increasing hydrogen content. Such behavior is also confirmed by soft X‐ray magnetic circular dichroism (XMCD) measurements as shown in the inset of Figure 4c.^25^ Our theoretical calculations suggest that all HSCO phases possess a G‐type antiferromagnetic magnetic ground state (Table S2, Supporting Information), and the spontaneous magnetization could be attributed by the canted moment resulting from the Dzyaloshinsky–Moriya (D–M) interactions,^26^, ^27^ which is strongly correlated with the local lattice distortion. If the Co—O—Co angle deviates from 180° (i.e., O is no longer the inversion center), the D–M interaction would emerge,^27^, ^28^ and lead to a canted moment through the competition with the Heisenberg interaction. Thus, using the deformed Co—O—Co bonding angle (180‐θ) (lower panel of Figure 4d) as an indicator for the lattice distortion, the theoretically calculated values indeed show nicely a consistent trend with the experimental observed in‐plane remnant magnetization (*M*_r_) as demonstrated in the upper panel of Figure 4d. Therefore, the comparison suggests that the increasing expansion of the lattice structure from HSCO‐A to HSCO‐B and then HSCO‐C would lead to the suppression of both the lattice distortion and the canting magnetization.

## 3 Conclusion

To summarize, using gating voltages as a chemical potential tuning knob in this newly developed ILG method, we demonstrated that it is possible to tune phase transformations in the model system of SrCoO_2.5_ and HSrCoO_2.5_ through controllable hydrogen evolution. We discovered two additional, yet unexplored phases with dramatically enhanced hydrogen concentrations. Our results reveal the formation of unexpected H–H dimers within the lattice, which leads to a systematic modification of the crystalline structure as well as the electronic and magnetic states, despite the unchanged Co valence state. The discovery of exotic H–H dimer in complex oxides, with a charge neutral chemical state, might hold great potential in the fields of energy storage, iontronics, etc. We would like to further point out that similar to the biased voltage, other external perturbations, such as temperature and pressure, might be equally important to tune the chemical potential difference and thus form a rich platform to rationally manipulate the phase transformation during ionic evolutions.

## 4 Experimental Section

*Synthesis of SrCoO_2.5_ and H_x_SrCoO_2.5_ Thin Films*: Epitaxial SrCoO_2.5_ thin films (≈40 nm) were grown on Nb:SrTiO_3_ (001) (0.5 wt%) and SrTiO_3_ (001) substrates using a home‐built pulsed laser deposition systems. The growth condition was optimized at 750 °C and oxygen atmosphere of 100 mTorr. The laser energy (KrF, λ = 248 nm) was kept at 1.2 J cm^−2^ with a repetition rate of 2 Hz. After deposition, the samples were cooled down to room temperature at the cooling rate of 7 °C min^−1^ in 100 mTorr to avoid overoxidation. The crystalline structure of the SrCoO_2.5_ thin films were characterized by X‐ray diffractometer (Rigaku, Smartlab) and the reciprocal space mappings (RSM) were collected at beamline 1W1A of Beijing Synchrotron Radiation Facility. The HSCO phases were fabricated as illustrated in Figure S1c in the Supporting Information, using commercial, as‐received ionic liquid (DEME‐TFSI).

*In Situ XRD*: The in situ XRD measurements were performed on the same instrument (XRD, Rigaku, Smartlab) as used to characterize the as‐grown samples. The SrCoO_2.5_/SrTiO_3_ thin films were chosen for the conventional ILG measurement. Conducting silver paint at the edges of the samples was used as working electrode (WE). For the newly developed ILG technique, the SrCoO_2.5_/Nb:SrTiO_3_ thin films were used and the conducting substrate (Nb:SrTiO_3_) was employed as working electrode. In both setups, platinum coils (≈25 turns) were used as the counter electrode (CE). Sample and CE were both immerged into a small quartz tank filled with a small amount of ionic liquid. To trace the structural evolution during the ILG, the gating voltage was set to zero at the beginning (≈10 min), and then quickly increased to the desired voltages within 15 s, while the XRD scans were performed continuously with the scanning rate of 1–3° min^−1^.

*High‐Resolution STEM*: For STEM, all samples were prepared using a focused ion beam (FIB) instrument. It was noted that the sputtering process of Pt protection layer during the TEM sample preparation would cause notable heating effect to the sample, which would be the dominated mechanism leading to the hydrogen loss due to the thermal stability of the HSCO samples (Figure S13, Supporting Information). To avoid this, the sputtering time for the Pt layer growth was limited to only ≈10 s and then repeated six cycles with interval of 3 min, which would lead to the growth of total 40 nm Pt as the capping, which can nicely keep the hydrogen stable in the thin film during the sample preparation. For STEM image in main text Figure 3, the samples were thinned down to 50 nm using an accelerating voltage of 30 kV with a decreasing current from 240 to 50 pA, followed by fine polish with an accelerating voltage of 5 kV with current of 20 pA. The HAADF and ABF images were acquired on an FEI Titan Cubed Themis 60‐300 (operated at 300 kV), which is equipped with a high brightness electron gun (X‐FEG with monochromator), a Cs probe corrector, and a Cs image corrector. The convergence semiangle for STEM‐HAADF and STEM‐ABF imaging was 25 mrad, while the collection angles of HAADF and ABF detector were 64–200 and 8–13 mrad, respectively. The simulation of HAADF and ABF images was carried out with the same semi‐convergence angle and semi‐collection angle to experiments using multislice methods by the xHREM software (developed by Ishizuka). A unit cell was divided into 10 slices and the thickness was set to 10 nm.

*Soft X‐Ray Absorption Measurements*: The sXAS data of Co *L*‐edge and O *K*‐edge were collected in the total electron yield (TEY) mode at beamlines 4.0.2 and 6.3.1 of Advanced Light Source and beamline 4B9B at Beijing Synchrotron Radiation Facility. The experiments were performed under high vacuum of 10^−8^ Torr at room temperature and the signal was normalized by the photocurrent from a clean gold mesh. The XMCD measurements were carried out at beamline 6.3.1 of Advanced Light Source, with incident angles of 30° from the surface. Magnetic field of 2 T was applied along the beam incident direction, and 65% circular polarized X‐ray was adopted.

*Optical Transmittance Measurements*: Double‐sided polished and transparent SrTiO_3_ (001) substrates were employed for the study and a thin (≈6 nm) conducting layer of SrRuO_3_ was deposited on the substrate to serve as the working electrode, while the SrRuO_3_ (≈6 nm) grown directly on SrTiO_3_ was employed as the reference for the background signal subtraction. The (ultraviolet–visible) UV–vis spectra, the ex situ optical transmittance spectra with wavelength from 190 to 3300 nm were obtained at room temperature in air by a Cary 5000 UV–vis–NIR spectrophotometer.

*First‐Principles Calculations*: Density function theory (DFT) calculations were performed using the Vienna ab initio simulation package (VASP 5.3.3). Projector‐augmented wave (PAW) potentials as parameterized by Perdew–Burke–Ernzerhof within the generalized gradient approximation (GGA) were used to account for electron exchange and correlation.^29^ The projector augmentation wave potentials included ten valence electrons for Sr (4s^2^4p^6^5s^2^), nine for Co (3d^8^4s^1^), and six for oxygen (2s^2^2p^4^). Typical computational parameters for the calculations were a 500 eV plane‐wave energy cut‐off, a (10 × 10 × 6) Monkhorst‐Pack *k‐*point sampling mesh set with respect to the perovskite supercell (containing eight chemical formula units of SrCoO_2.5_), and a 0.01 eV Å^−1^ force tolerance on each atom for structural relaxation calculations. The in‐plane lattice constants were fixed to be that of the substrate, while the out‐of‐plane lattice constant was fully optimized. The strong correlation effects of cobalt *d* electrons were included by means of the LDA+U scheme with *U*_eff_ = 4.0 eV. The search for the reasonable structure of HSCO‐B and HSCO‐C is summarized in Note S2 in the Supporting Information. The G‐AFM configuration was energy‐favored in all (H)SCO phases (Table S2, Supporting Information). The stretching vibration frequency was determined by phonon calculations as implemented in the Phonopy code.^30^

## Conflict of Interest

The authors declare no conflict of interest.

## Supporting information

Supplementary
