# Supplementary material for: Electric Field–Controlled Multistep Proton Evolution in HxSrCoO2.5 with Formation of H–H Dimer
Source: Adv Sci (Weinh). 2019 Aug 15;6(20):1901432. doi: 10.1002/advs.201901432 (PMC6794722; doi:10.1002/advs.201901432)
Supplement: Supplementary file 1 — Supplementary [file ADVS-6-1901432-s001.pdf]

Copyright WILEY-VCH Verlag GmbH & Co. KGaA, 69469 Weinheim, Germany, 2019.

ADVANCED SCIENCE
Open Access

Supporting Information

for *Adv. Sci.,* DOI: 10.1002/advs.201901432

Electric Field–Controlled Multistep Proton Evolution in $H_xSrCoO_{2.5}$ with Formation of H–H Dimer

Hao-Bo Li, Feng Lou, Yujia Wang, Yang Zhang, Qinghua Zhang, Dong Wu, Zhuolu Li, Meng Wang, Tongtong Huang, Yingjie Lyu, Jingwen Guo, Tianzhe Chen, Yang Wu, Elke Arenholz, Nianpeng Lu, Nanlin Wang, Qing He, Lin Gu, Jing Zhu, Ce-Wen Nan, Xiaoyan Zhong, Hongjun Xiang,* and Pu Yu**

Supporting Information Figures and Notes

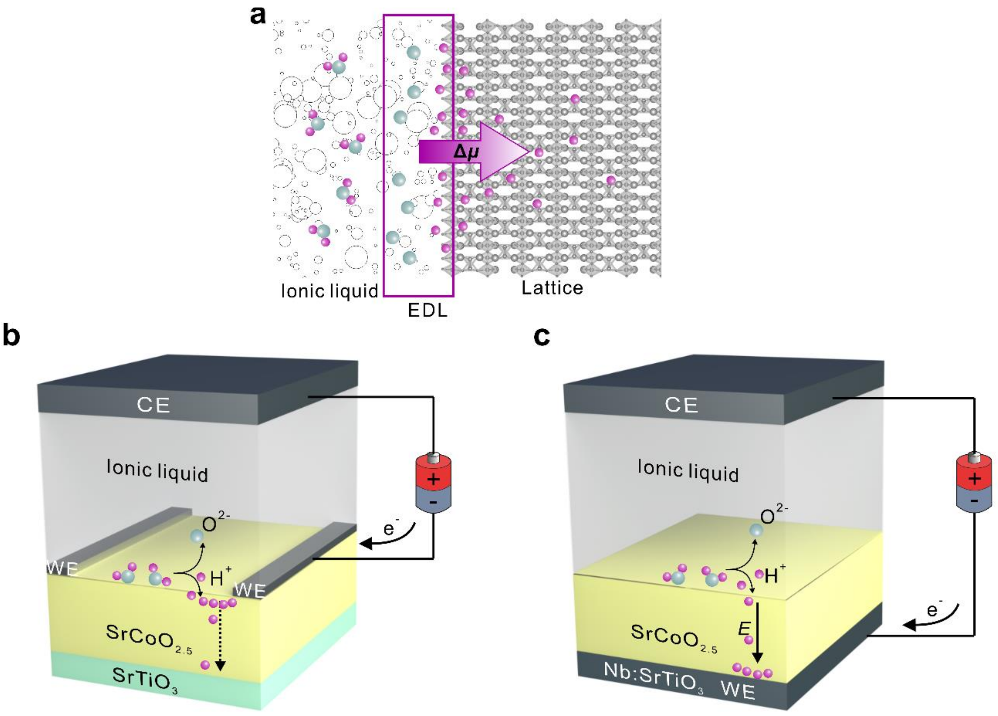

=

Figure S1. Proposed mechanisms for the electric-field controlled proton evolution through ILG. (**a**) Schematic drawing for ionic liquid gating induced protonation. The residual H_2O within the ionic liquid is electrolyzed into O^{2-} and H^+, and the positively charged H^+ ions are accumulated on the film surface due to the positive electric field and then injected into the thin film to form the protonated phase. The diffusion process is driven by the chemical potential difference $\Delta\mu$ across the sample surface. (**b**) Experiment setup for the conventional ionic liquid gating device. Platinum coils and silver paint serve as counter electrode (CE) and working electrode (WE), respectively. To form protonated $SrCoO_{2.5}$ phases, a positive voltage is applied between the CE and WE. (**c**) Chemical difference controlled ionic gating experimental setup with the WE underneath the thin film. This setup provides an electric field through the film to form the controllable parameter for the ionic liquid gating induced protonation.

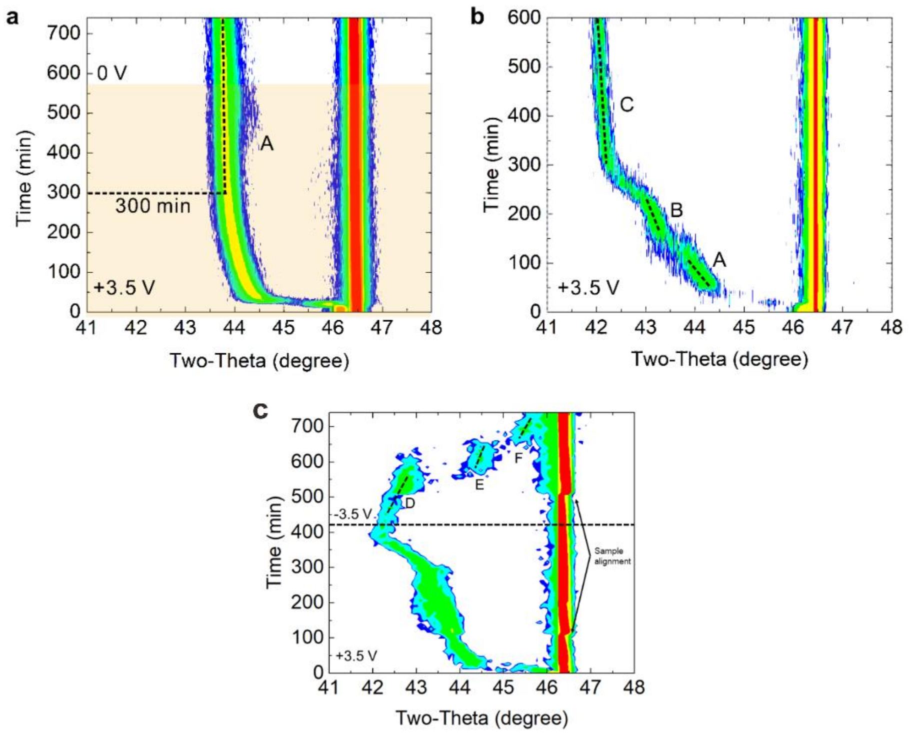

Figure S2. Extended *in-situ* XRD experiments. (**a**) ILG with conventional methods on $SrCoO_{2.5}/SrTiO_3$ as demonstrated in **Figure S1b.** With gating voltage of +3.5 V, the $SrCoO_{2.5}/SrTiO_3$ thin film shows the formation of a protonated phase (phase A), which remains stable even for extended gating period (**b**) ILG with modified methods as demonstrated in **Figure S1c**. No more phase transition occurs after the HSCO-C even with prolonged gating duration. (**c**) With the reversed bias voltage of -3.5 V, we have observed a series of structural transformation toward to the brownmillerite phase. First, the diffraction peak of HSCO-C moves continuously from 42.2° to 42.8° (phase D), indicating a gradually reduced out-of-plane lattice constant resulted from loss of hydrogen. Then a sharp phase transition occurs at about 44.5° (phase E). Finally, we obtained a final state at 45.7° (phase F), which would be in accord with the BM-SCO.

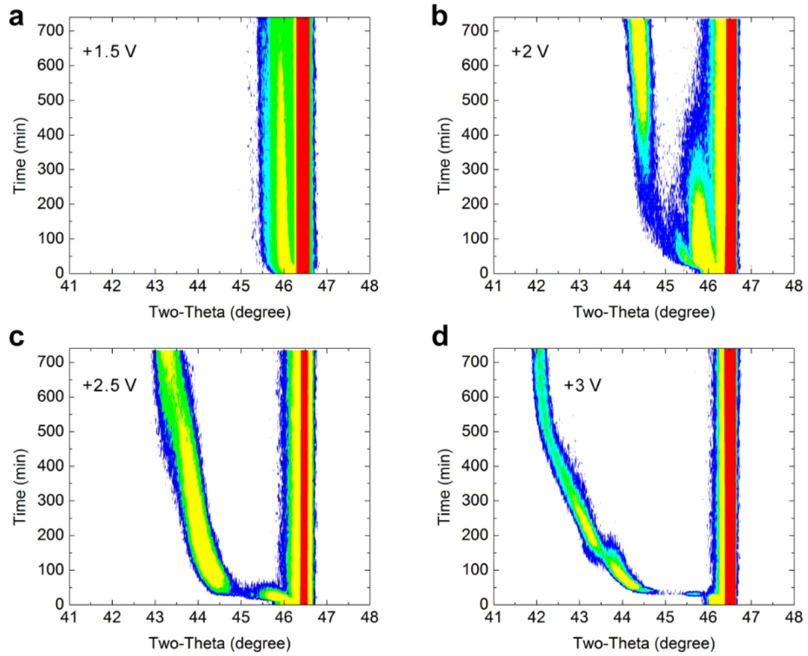

Figure S3. Electric-field controllable proton evolutions. Time dependent *in-situ* XRD scans around $SrCoO_{2.5}$ pseudo-cubic (002) peak with the gating voltages of (**a**) +1.5 V, (**b**) +2 V, (**c**) +2.5 V and (**d**) +3 V, respectively.

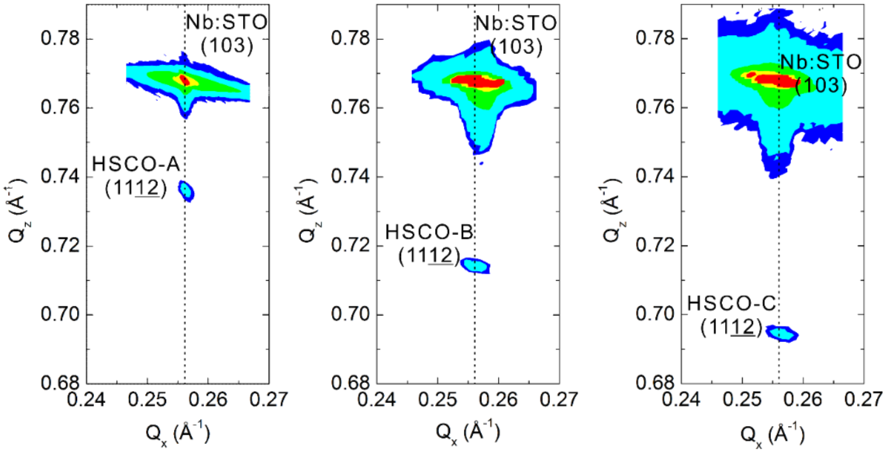

Figure S4. Reciprocal space mapping (RSM) results for different protonated $SrCoO_{2.5}$ phases. While all these HSCO phases are expanded along *c*-axis, they are coherently locked by the substrate with the epitaxial strain maintained.

Supporting Information Note 1: Compositional analysis of protonated $SrCoO_{2.5}$

Since the ILG can lead to both hydrogen and oxygen evolution and thus change the corresponding H and O stoichiometry, several methods were employed to provide quantitative estimates of the chemical composition in different HSCO phases. Energy-dispersive X-ray spectra (EDS) were collected to estimate the oxygen stoichiometry, during which the electron acceleration voltage is optimized to 4.5 kV to minimize the signal contribution from the substrate[1]. Then the O/Co ratio was roughly estimated by calculating the peak ratio between the oxygen K_{α} and cobalt L_{α} peaks. The obtained data in BM-SCO (with O/Co ~2.6) is well consistent with the oxygen stoichiometry of nominal $SrCoO_{2.5}$. While for all the HSCO phase, the O/Co ratios were estimated as 2.6±0.1 (**Figure S5a-c**), indicating the change of oxygen contents during the ILG was negligible.

To obtain direct information about the hydrogen concentrations within the HSCO phases, time of flight secondary-ion mass spectrometry (TOF-SIMS) was performed (IONTOF GmbH), where the mass resolution is 4000 atomic mass units (full-width at half maximum). During the study, each HSCO sample was bombarded with cesium-ion beams (2 keV) over a region of ~250×250 μm^2, while the data were only collected from central region of 50×50 μm^2 to avoid contamination. The concentration of H^+ was calibrated based on a proton-implanted silica reference sample with known hydrogen concentration (~1.6×10^{17} atoms/cm^3). From these studies, we estimated that the hydrogen concentrations for HSCO-A, HSCO-B and HSCO-C were 1.27×10^{22}, 2.18×10^{22} and 2.81×10^{22} atoms/cm^3 respectively. We further deduced the hydrogen concentration using the volume size of the pseudo-cubic lattice constant calculated from XRD results, where the unit cell volumes of HSCO-A, HSCO-B and HSCO-C were 6.84×10^{-22}, 7.41×10^{-22} and 7.95×10^{-22} cm^3 respectively. With that, we estimated the atomic ratios of H/Co in these phases are 0.9, 1.6 and 2.2 with an uncertainty of ~±0.1. We note that the deviation from the actual value could be larger due to different matrix elements for SIMS studies between the sample and the reference.

To provide a comparison study, we also carried out the hydrogen forward scattering spectrometry (HFS) analysis (**Figure S5d**), which is consistent nicely with the SIMS

results. During the HFS measurement, the detector was placed 30° from the forward trajectory of the incident He^{++} ion beam and a thin absorber foil was placed over the detector to filter out the forward scattered He^{++} ions, whose energy was 2.275 MeV.

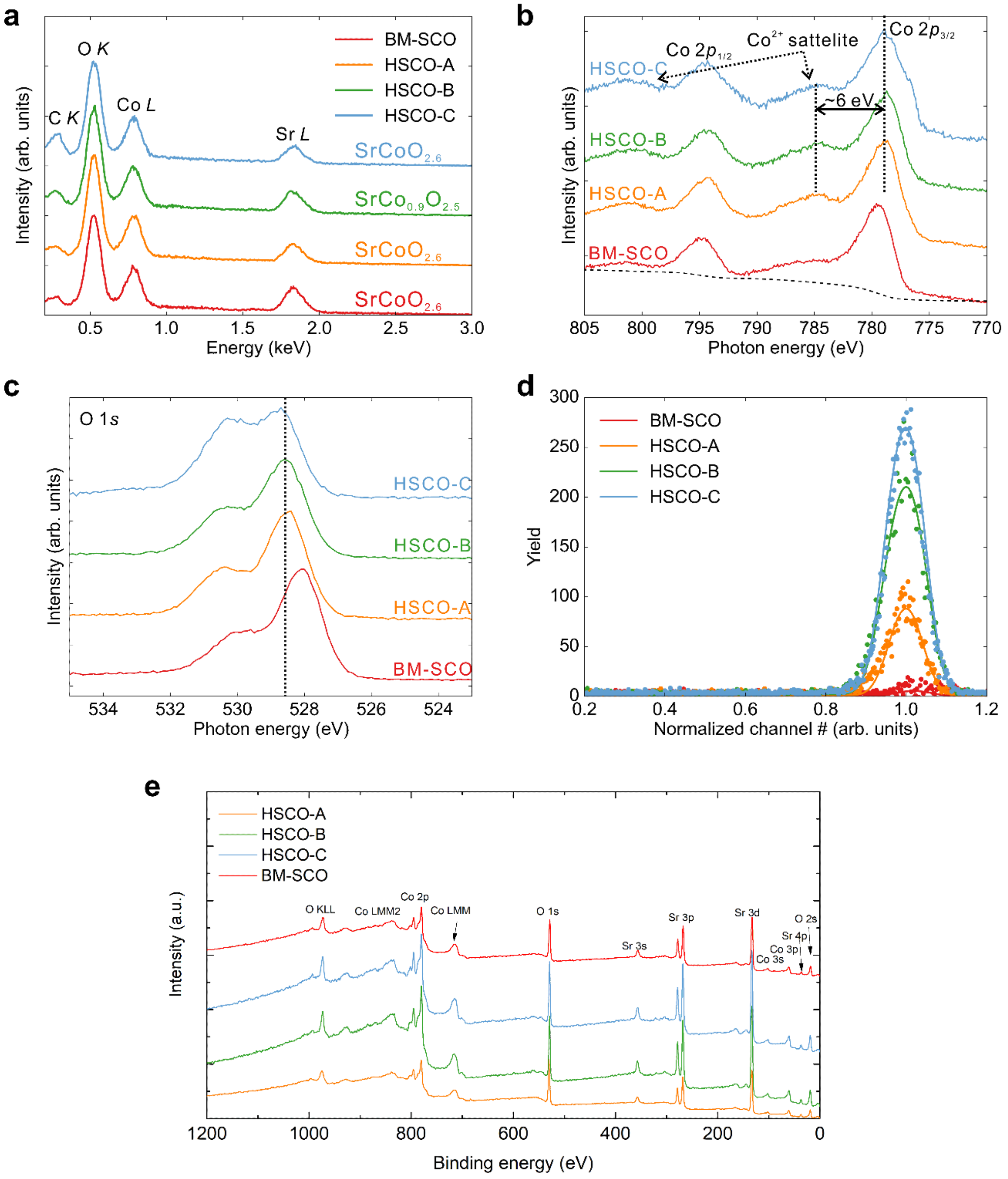

Figure S5. Extended chemical compositional analysis for all HSCO phases. (a) Energy dispersive spectra (EDS) of three HSCO phases as well as the reference BM-SCO.

The optimized acceleration voltage of 4.5 kV was used during the measurements in order to minimize the signal from the substrate[2]. The O/Co ratios are roughly estimated by calculating the peak ratio between the oxygen K_α and cobalt L_α peaks, where all these phases possess similar O/Co ratio of 2.6:1. X-ray photoelectron spectra at (**b**) Co *2p* and (**c**) oxygen 1*s* states of all phases. In Co 2*p* spectra, the Co $2p_{3/2}$ peaks of HSCO phases shift about ~0.7 eV toward the lower energy as comparing with that of the BM-SCO, indicating the reduced Co valence state. Besides, the enhanced Co^{2+} satellite peaks suggest the existence of Co^{2+} valency in all HSCO phases. For the O 1*s* spectra, the binding peaks shift to higher energy in all HSCO phases, consistent with the typical feature observed when O-H bond is formed[3]. The atomic ratio of O/Co was obtained by calculating the area ratio between O 1*s* and Co 2*p*, which is around 2.5±0.1:1 for all three HSCO phases. (**d**) Hydrogen forward scattering spectra (HFS) for HSCO samples. According to the Gaussian fittings of experimental data, the H to Co concentration ratios were estimated as 0.7, 1.6 and 1.9 for HSCO-A, HSCO-B and HSCO-C, respectively. (**e**) The XPS full scan indicates that only Sr, Co, O are detectable, which guarantees that no side production is created in the thin film during the ILG.

Supporting Information Note 2: Evidence of H-H dimer within protonated $SrCoO_{2.5}$

To obtain further atomic structural insights into the protonated phases, we have performed aberration-corrected scanning transmission electron microscopy (STEM) studies. The high-angle angular dark-field (HAADF) STEM images (**Figure S6a-d**) show clearly alternating stacking of octahedral CoO_6 and tetrahedral CoO_4 sub-layers as well as the Co-Co dimerization in the tetrahedral layers for BM-SCO and all HSCO phases. This indicates the robustness of the brownmillerite crystalline framework even with the intercalation of extensive hydrogen contents. Furthermore, line scans in the HAADF images (yellow dashed lines in **Figure S6a-d**) reveal that the out-of-plane pseudo-cubic lattice constants are 3.93, 4.10, 4.21 and 4.31 Å in average for BM-SCO, HSCO-A, HSCO-B and HSCO-C phases respectively, which is consistent nicely with our XRD results. The local Sr-Sr interatomic distance in octahedral CoO_6 layers (d_{oct}) and tetrahedral CoO_4 (d_{tet}) layers are summarized in main text **Figure 3b**.

Knowing both the chemical composition as well as basic crystalline framework of the HSCO phases, we performed density functional theory (DFT) calculations to determine the possible crystalline structures for these new phases (See **Supporting information Note 3** for details). The optimized structures of BM-SCO, HSCO-A, HSCO-B and HSCO-C are illustrated in **Figure S6e-h**, respectively, showing the obtained lowest energy configuration as well as representative higher-energy configurations. The calculation reveals that the hydrogens within HSCO-A (**Figure S6f)** bond with the interlayer oxygen ion sandwiched between tetrahedral and octahedral sub-layers where half of O-H bonds head to the tetrahedral sub-layer and the other half towards the octahedral sub-layer. In this configuration, the formation of O-H bond competes with the nearest Co-O bond and leads to the formation of reduced Co^{2+} as well as suppressed *p-d* hybridization, agreeing nicely with the experiment results. Surprisingly when additional hydrogen ions are inserted into the crystalline structure to form HSCO-B and HSCO-C phases, the hydrogen atoms form naturally the H-H dimer located in the original ordered oxygen channels to minimize the total energy of the system (**Figure S6g and h**). The calculations also reveal that the orientation of H-H dimer tends to maintain along the in-plane direction. Furthermore, the selection of oxygen channels containing

the H-H dimers has little impact on the total energy, suggesting the H-H dimers might be randomly distributed within the lattice especially for the case of HSCO-B. Moreover, these H-H dimers have negligible interaction with surrounding oxygen atoms, implying that the valence state of Co and the oxygen related hybridization with other elements cannot be further modulated when HSCO-A is transformed into HSCO-B and HSCO-C, which also well accords with the experimental XAS results. It is worth noting that during the phase transformation from HSCO-B to HSCO-C, the orientation of the O-H bond rotates from the tetrahedral sub-layer toward the octahedral sub-layer (**Figure S6h**), resulting in the unique transition of the interplanar distance of both d_{tet} and d_{oct} as illustrated in main text **Figure 3b**.

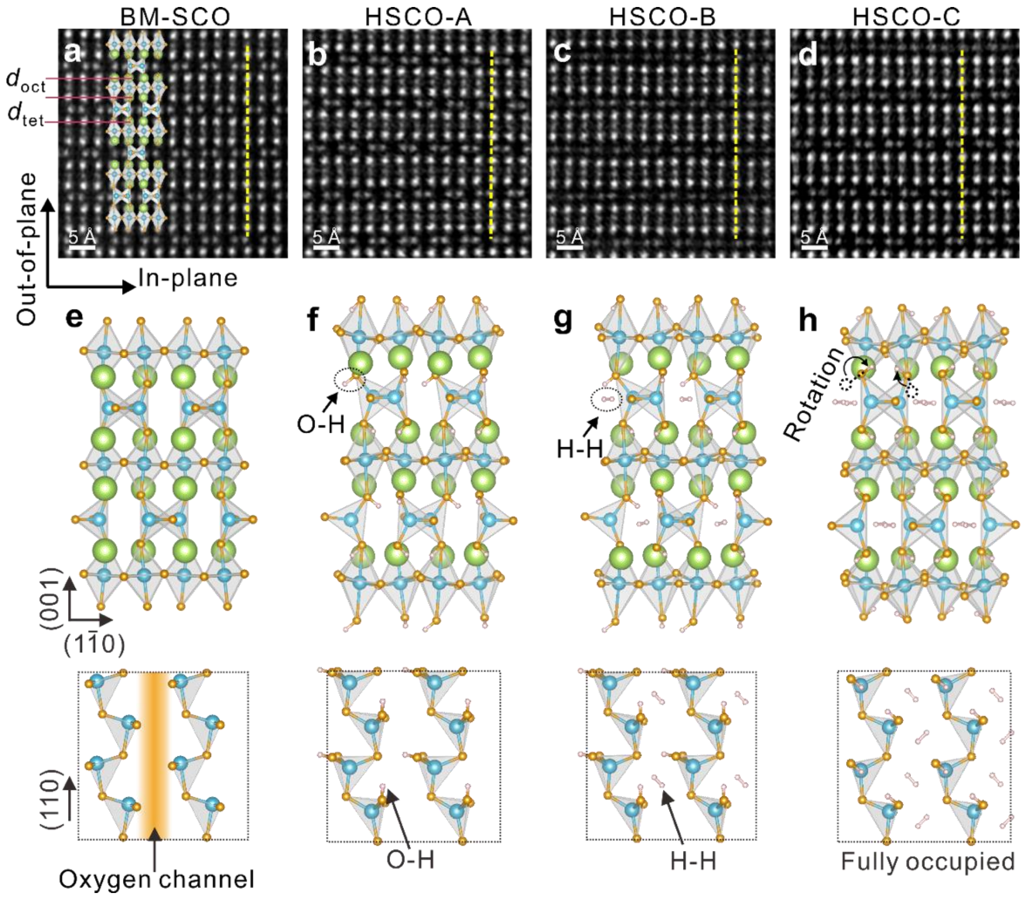

Figure S6. Comparison of experimental structural characterizations and theoretical calculations for HSCO phases. For the STEM images in this figure the samples were thinned down to 100 nm using an accelerating voltage of 30 kV with a decreasing current from the maximum 2.5 nA, followed by fine polish with an accelerating voltage of 2 kV

with current of 40 pA. The images were obtained with the transmission electron microscope equipped with double spherical aberration (Cs) correctors (ARM-200CF, JEOL, Tokyo, Japan). High-angle annular dark field (HAADF) scanning transmission electron microscopy images of (**a**) BM-SCO, (**b**) HSCO-A, (**c**) HSCO-B and (**d**) HSCO-C along the (110) projection of the Nb:$SrTiO_3$ (001) substrates. (**e-h**) Calculated atomic crystalline structure of corresponding HSCO phases. The top and bottom rows show the results for side-view from (110) and top-view from (001) directions, respectively. In the top view figures, only the CoO_4 tetragonal layers are displayed for clearance purpose. Experimental results for the interlayer distances between Sr ions separated by CoO_4 ($d_{Sr\text{-}tet}$) and CoO_6 ($d_{Sr\text{-}oct}$) are obtained along the yellow lines in (**a-d**) and summarized in **Figure 3b**. The error bars indicate the standard deviation of the data.

Supporting Information Note 3: Theoretical calculations of the crystalline structures of protonated $SrCoO_{2.5}$

To simulate the structures for HSCO-A ($H_{0.9}SrCoO_{2.5}$), HSCO-B ($H_{1.6}SrCoO_{2.5}$), and HSCO-C ($H_{2.2}SrCoO_{2.5}$), three nominal compositions of $H_{1.0}SrCoO_{2.5}$, $H_{1.5}SrCoO_{2.5}$, and $H_{2.0}SrCoO_{2.5}$ were adapted. Accordingly, we randomly inserted eight, twelve and sixteen hydrogen atoms into the $SrCoO_{2.5}$ (*Ima2* spacegroup) supercell consisting of eight chemical formula units, and then optimized the structures to search for the lowest energy configurations. To mimic the epitaxial strain for all $H_xSrCoO_{2.5}$ phases, the in-plane lattice constants were fixed to that of the STO substrate, while the out-of-plane constant was allowed to relax during the calculations.

For $H_{1.0}SrCoO_{2.5}$ phase, our calculations show that the eight hydrogen atoms tend to bond with the interlayer oxygen atoms sandwiched between the tetrahedral and octahedral sub-layers, in agreement with previous calculated results[4]. However, in $H_{1.5}SrCoO_{2.5}$, the structures with two H-H dimers are energetically more stable than the other structures. As typical examples, four optimized structures are shown in **Figure S7**. These structures differ in the way that where the additional four hydrogen atoms occupy. **Figure S7a** contains two H-H dimers which occupy the oxygen-deficient channels; In **Figure S7b and S7c,** four hydrogen atoms are absorbed on the intra-layer oxygen atoms in the tetrahedral or octahedral sub-layers, respectively; In **Figure S7d**, two hydrogen atoms are absorbed in the tetrahedral sub-layers and the other two hydrogen atoms locate in the octahedral sub-layers. Three interesting observations can be obtained from the calculations. First, the absorption of H atoms into the intra-layer oxygen atoms of the tetrahedral or octahedral sub-layer would lead to the breakdown of polyhedral frameworks in the tetrahedral or octahedral sub-layers. Secondly, the four additional H atoms can form two H-H dimers to occupy the oxygen-deficient channel. Thirdly, the structures of $H_{1.5}SrCoO_{2.5}$ with two H-H dimers are energetically more stable than the other structures (we also found that the most stable structure for $H_{1.25}SrCoO_{2.5}$ contains one H-H dimer).

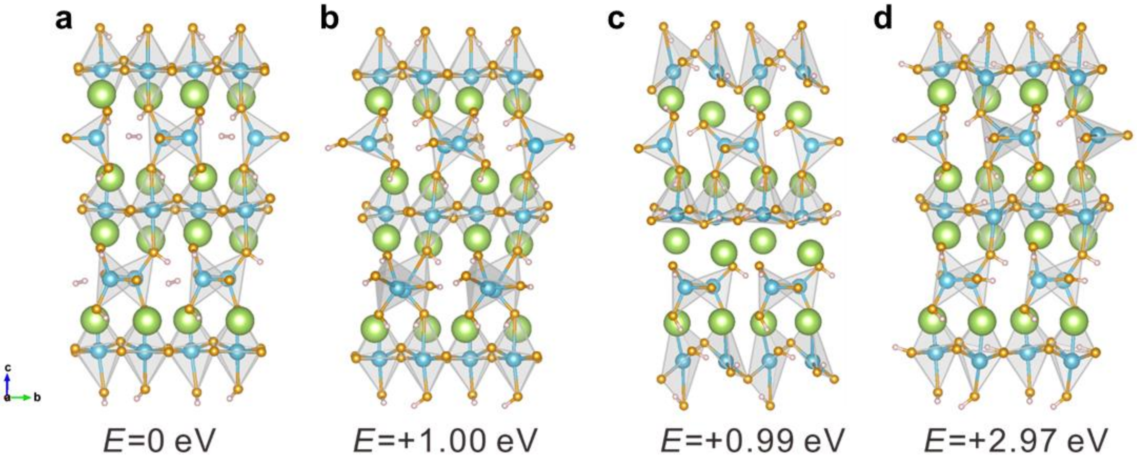

Figure S7. Calculated crystalline strutures for $H_{1.5}SrCoO_{2.5}$ phase with the additional four hydrogen atoms occupying different locations. (**a**) Calcualted cystalline structure contains two H-H dimers; in (**b**) and (**c**) four hydrogen atoms are absorbed on the intra-layer oxygen atoms in the tetrahedral or octahedral sub-layers, respectively; in (**d**) two hydrogen atoms are absorbed in the tetrahedral sub-layers and the other two hydrogen atoms in the octahedral sub-layers. Obviously, the formation of H-H dimers will lower the total energy, while the hydrogens absorbed on the intra-layer oxygens will disrupt the CoO_4 or CoO_6 polyhedral frameworks.

For the $H_{2.0}SrCoO_{2.5}$, our calculations suggest that the structure with four H-H dimers occupying the four oxygen-deficient channels is energetically more stable than other configurations (**Figure S8**). The extra hydrogen ions bonding with oxygen ions can cause instability of the systems, some of which are not able to energetically converged, indicating the formation of H-H dimer is crucial to stabilize the system.

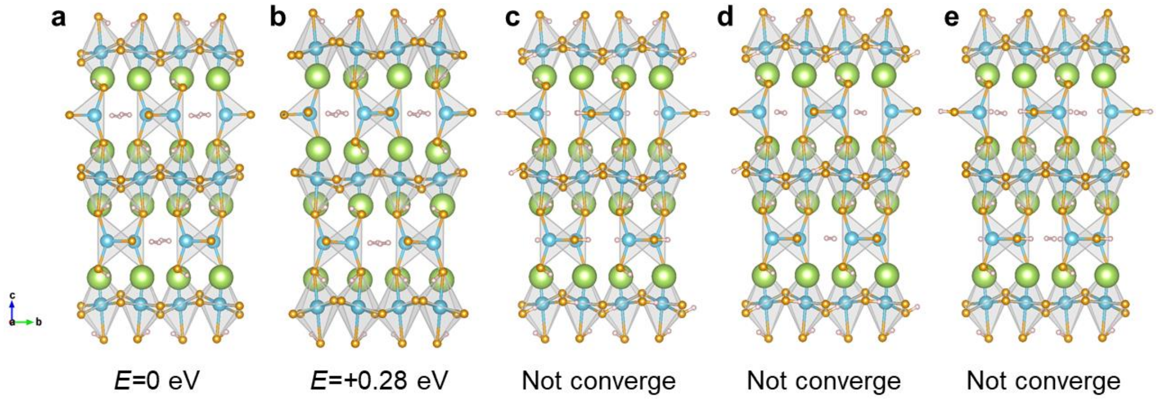

Figure S8. Calculated crystalline strutures for $H_{2.0}SrCoO_{2.5}$. (**a, b**) $H_{2.0}SrCoO_{2.5}$ with oxygen channel fully occupied by H-H dimer. $H_{2.0}SrCoO_{2.5}$ with (**c**) totally empty and (**d, e**) half-filled oxygen channels and the rest H ions are bonding with octahedral oxygens. It is worth noting that the total energy of the system is sensitive to the O-H bonding directions. When the oxygen channel is fully occupied, the structures with O-H bonding towards the CoO_4 tetragonal layer are all unstable. Even when the O-H bonds point to the CoO_6 layer as demonstrated in (**a**) and (**b**), their exact directions can also influence the total energy of the system. Thus after careful investigation, we find (**a**) is the structure with proper O-H bonding direction that can minimize the total energy.

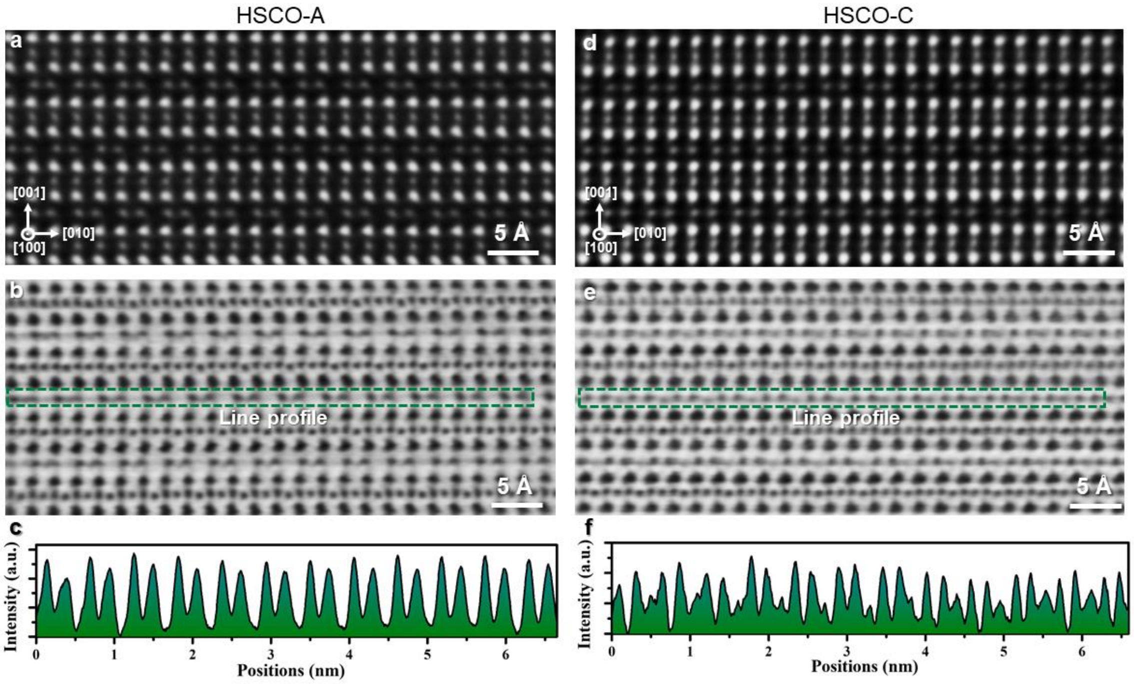

Figure S9. Comparision of STEM measurements for HSCO-A and HSCO-C. (a) HAADF-STEM and (**b**) ABF-STEM images of HSCO-A, in which feature of H-H dimer is absent. (**c**) Line profile along the selected area in (**b**). (d) HAADF and (e) ABF images of HSCO-C. The H-H dimers are orderly located in the lattice, which agree with the theoretical prediction. (**f**) Line profile in the selected region in (**e**).

Supporting Information Note 4: Spectral evidence of the H-H dimers

To further verify the presence of O-H bonds and H-H dimers within protonated phases, we also carried out infrared (IR) spectra measurements for BM-SCO, HSCO-A, HSCO-B, and HSCO-C. As shown in **Figure S10**, except BM-SCO, all IR spectra exhibit absorption feature at ~3460-3500 cm^{-1}, which can be attributed to the characteristic stretching mode in O-H groups[5]. Considering the fact that in H_2 molecular, a close analog to the H-H dimer in our systems, the symmetric vibration in stretching mode is IR-inactive[6,7], we introduced a partial deuterium (D) substitution by doping heavy water into the ionic liquid, which can then generate the required dipole moment change for the IR measurements with H-D groups, in which the mass difference between H and D atoms would introduce a displacement between the centers of the electronics and nuclear charges, then leading to the formation of dipole moment[8,9]. Clearly, the resulting H(D)SCO-B and H(D)SCO-C samples show the emergence of a unique absorption feature at frequency slightly lower than the O-H absorption peak, as compared with the undoped HSCO-C and such absorption is weaker in H(D)SCO-B than H(D)SCO-C, which is associated with the hydrogen content in these two phases. Indeed, the theoretical calculations predict that the H-D characteristic frequency in H(D)SCO-C is about ~67-134 cm^{-1} lower than that of the O-H absorption (**Table S1**). Furthermore, when more D_2O is doped into the ionic liquid, the intensity of the H-D absorption can be greatly enhanced (**Figure S11**), which further verifies the existence of H-D dimers.

We note that the reported fingerprint vibration frequency for H-D is around ~3200 cm^{-1} in semiconductors[10,11], which is ~200 cm^{-1} lower than that observed in our current system. We speculate that the energy shift might be attributed to the different local chemical environments dominated by the neighbor ions[12]. In our system, the O-2*p* XAS spectra (main text **Figure 2c**) indicate that the feature of the O-Co hybridization is nearly unchanged among HSCO-A, B and C, which suggests that the H-H dimer may have relatively weaker interaction with their neighbor ions than that of the H-D in silicon. This may be the origin of the slightly increased vibration frequency of H-D dimers in our systems. It is also worth noting that a weak absorption at ~2180 cm^{-1} can also be observed in **Figure S11**, which can be assigned as the stretch vibration of O-D group.

The infrared (IR) transmittance measurements were obtained with a Bruker 80v/S spectrometer. The measurements were performed in vacuum with background of ~120 Pa and the spectra were obtained in wavenumber ranging from 1500 to 6000 cm-1 at room temperature. To introduce deuterium substitutions, the H_2O and D_2O mixture (with the ratio of 1:1) was slowly added into the DEME-TFSI until the volume ratio reached 1:100. After stirring for 30 minutes, the doped ionic liquid was baked under 120 °C for 48 hours to evaporate the extra H_2O and D_2O.

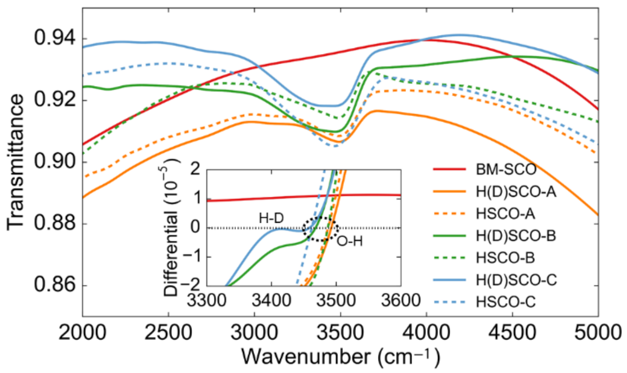

Figure S10. Room-temperature infrared spectra for BM-SCO and all three HSCO phases. To obtain clear feature from the dimers, we used deuterium to replace partial hydrogen to form the infrared-active H(D)SCO. The inset shows the differential analysis of the spectra, and the black circle denotes the strong O-H absorption in all protonated phases.

Table S1. Calculated stretching vibration frequencies (cm^{-1}) of O-H and H-D bonds in H(D)SCO-C. The calculations were carried out at T=0 K, while distinct energy shift might be obtained at higher temperature (i.e., room temperature) conditions.

	Min	Max	Average
O-H	3136	3269	3202
H-D	2935	3202	3068
O-D	2268	2401	2335

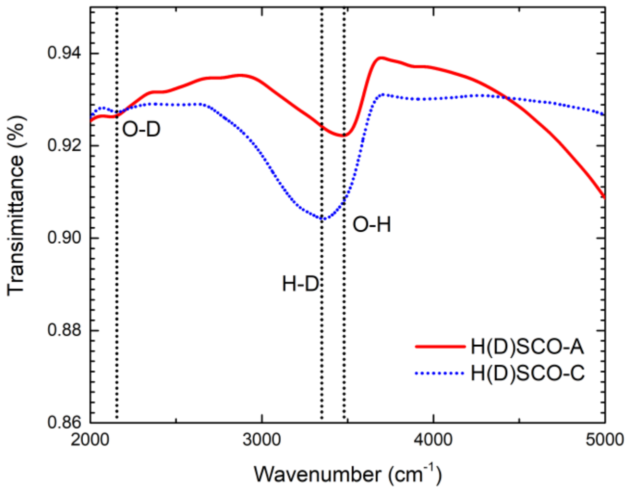

Figure S11. Infrared spectra of heavly deterium doped HSCO phases. To enhance the deterium concentration, we used pure D_2O as a dopont into the ionic liquid. As a consequence, the H-D absorption characteristic peak is greatly enhanced in the H(D)SCO-C phase due to the enhanced deterium concentration. It is worth noting that due to the unavoidable H_2O residual within the ionic liquid, the characteristic absorption peak for O-H goups can also be observed in this case.

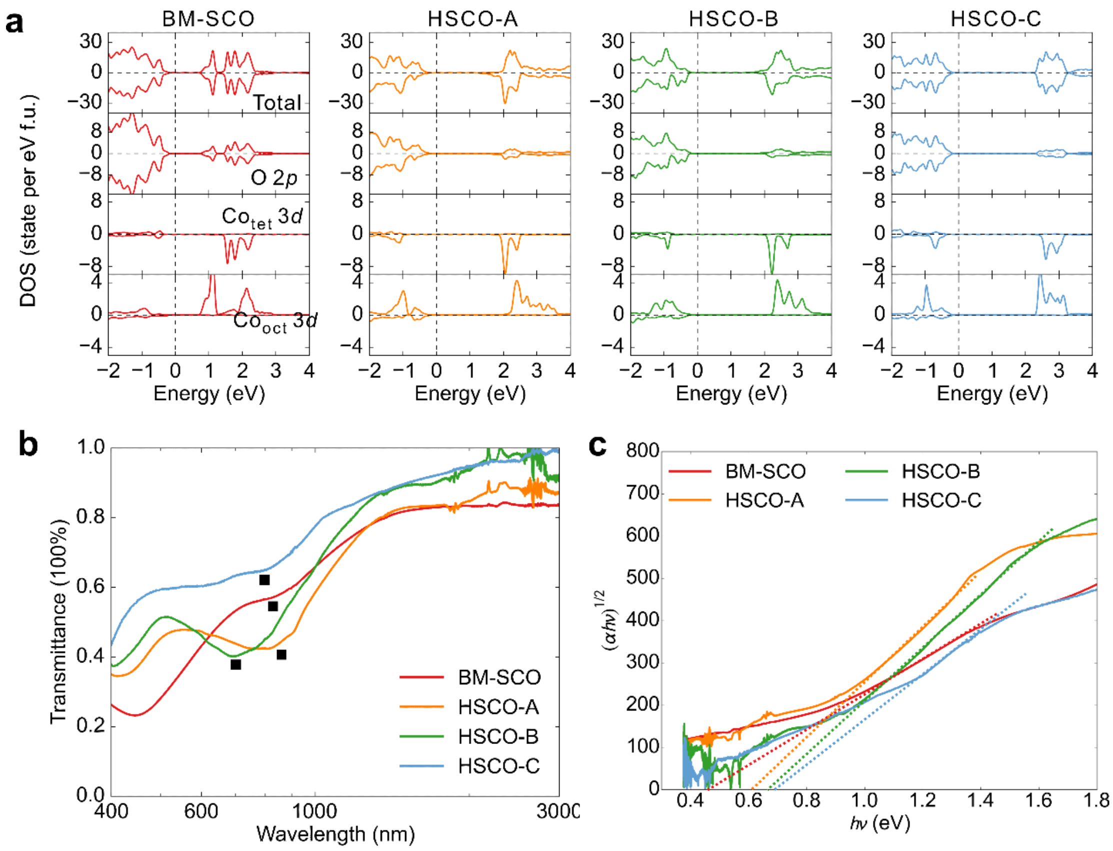

Figure S12. Evolution of the density of states and optical absorption spectra for BM-SCO, HSCO-A, HSCO-B and HSCO-C. (**a**) The calculations reveal a dramatic increase of the optical band gaps due to the ascension of conduction band along with the increasing proton concentrations, which agree nicely with the experimental observations. (**b**) Comparison of the absorption coefficients for the BM-SCO and HSCO phases. The black squares demonstrate the *d*-*d* transitions[13]. (**c**) The indirect optical band gap (E_g) are fitted by $(\alpha h\nu)^{1/2}=A(h\nu-E_g)$, where A is an energy-independent pre-factor. The inset demonstrates the direct optical band gap fitted by $(\alpha h\nu)^2=B(h\nu-E_g)$, where B is a pre-factor as well. The indirect band gap are fitted as ~0.48, ~0.61 ~0.65 and ~0.70 eV for BM-SCO, HSCO-A, HSCO-B and HSCO-C phase, respectively. As compared with BM-SCO, the *d*-*d* intra-band transition is enhanced in HSCO-A and B phases, while suppressed in HSCO-C. Such behavior might be attributed to the distortions of the CoO_4 and CoO_6 ligands after hydrogen injection, which modulates strongly the electron orbital overlapping and eventually influences the *d*-*d* transition in the oxides[14]. When increasing lattice distortions in HSCO-A and B, the Co-O-Co bonding angle is increased, which

eventually enhances the *d*-*d* transition. While in HSCO-C, the Co 3*d* states are more localized than other phases, hence the *d*-*d* transition is greatly suppressed. Furthermore, as point defects can also introduce local impurity bands within the band gap and may trigger additional optical transitions[15]. Therefore, the enhanced absorption intensity at the absorption edge in HSCO-B can also be related to the large amounts of defects during the phase transformation, in which the H-H dimmers are randomly distributed within the oxygen channels, leading to the formation of local defects and disorder in the material system.

Table S2. Calculated total energies (eV) of different magnetic states for all three HSCO phases and BM-SCO.

Phase	A-AFM	C-AFM	G-AFM	FM
BM-SCO	+0.883	+0.581	0	+1.251
HSCO-A	+0.460	+0.058	0	+0.521
HSCO-B	+0.430	+0.063	0	+0.499
HSCO-C	+0.374	+0.125	0	+0.517

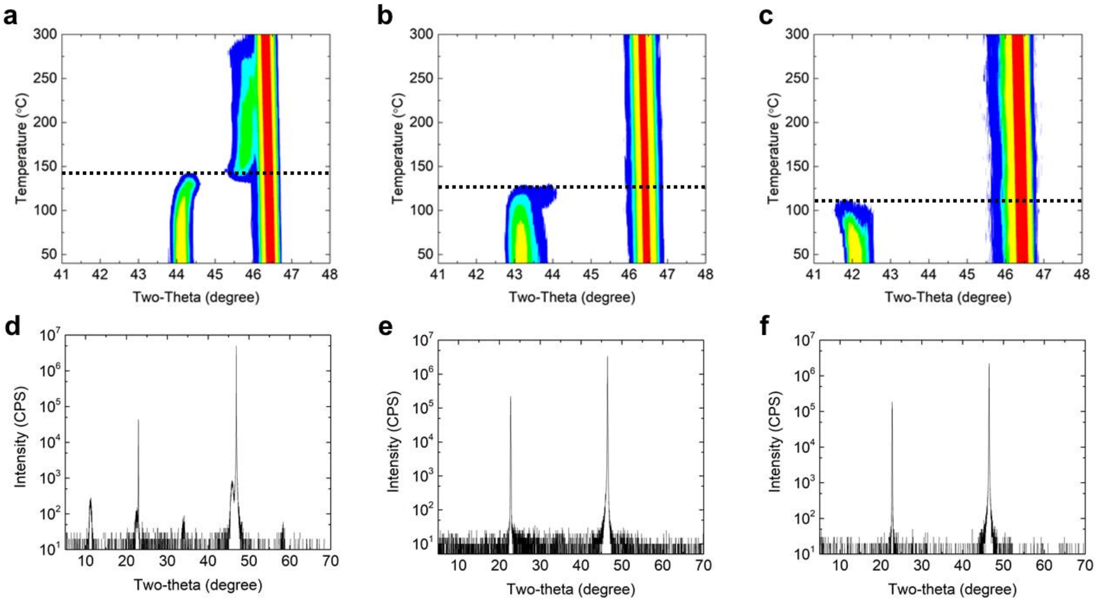

Figure S13. Thermal stability test of HSCO phases in air. Temperature dependent *in situ* XRD studies of the (a) HSCO-A, (b) HSCO-B and (c) HSCO-C phases, respectively. Full scan of the (d) HSCO-A, (e) HSCO-B and (f) HSCO-C annealed in air at 155, 140 °C and 130 °C, respectively. Consistent with the previous study[3], the HSCO-A phase remains stable up to ~140 °C, and then returns to BM-SCO at higher temperature with the featured superlattice peaks identified. Similarly, the HSCO-B and HSCO-C remain stable around room temperature, and then are completely decomposed at ~125 °C and ~115 °C respectively, with the totally disappeared structural XRD peaks. Meanwhile the full scans demonstrate that no other peaks can be observed except the substrate. These results suggest that the released H-H dimers under heating may be highly reductive, which would damage consequently the thin film during heating.

References

1. Katayama, T. *et al.* Topotactic synthesis of strontium cobalt oxyhydride thin film with perovskite structure. *AIP Advances* **5,** 107147 (2015).

2 Katayama, T. *et al.* Topotactic synthesis of strontium cobalt oxyhydride thin film with perovskite structure. *AIP Advances* **5,** 107147 (2015).

3 Dupin, J. C., Gonbeau, D., Vinatier, P. & Levasseur, A. Systematic XPS studies of metal oxides, hydroxides and peroxides. *Phys. Chem. Chem. Phys.* **2,** 1319-1324 (2000).

4 Lu, N. P. *et al.* Electric-field control of tri-state phase transformation with a selective dual-ion switch. *Nature,* **546,** 124-128 (2017).

5. G. Socrates, *Infrared and Raman Characteristic Group Frequencies: Tables and Charts*. John Wiley & Sons. **2012**, ISBN 978-0-470-09307-8.

6. A. Mainwood, A. M. Stoneham. *Physica B* **1983**, *116*, 101.
7. G. C. Van de Walle, Y. Bar-Yam, S. T. Pantelides, *Phys. Rev. Lett.* **1988**, *60*, 2761.
8. M. Trefler, H. P. Gush. *Phys. Rev. Lett.* **1968**, *20*, 703.
9. J. B. Nelson, G. C. Tabisz. *Phys. Rev. Lett.* **1982**, *48*, 1393.
10. Shi, G. A., Stavola, M., Fowler, W. B. & Chen, E. E. Rotational-vibrational transitions of interstitial HD in Si. *Phys. Rev. B.* **72,** 085207 (2005).
11. Chen, E. E., Stavola, M., Fowler, W. B. & Walters. P. Key to Understanding Interstitial H2 in Si. *Phys. Rev. Lett.* **88,** 105507 (2002).
12. Estreicher, S. K., Wells, K., Fedders, P. A. & Ordejón, P. Dynamics of interstitial hydrogen molecules in crystalline silicon. *J. Phys.: Condens. Matter* **13,** 6271-6283 (2001).
13. Choi, W. S. *et al.* Reversal of the Lattice Structure in $SrCoO_x$ Epitaxial Thin Films Studied by Real-Time Optical Spectroscopy and First-Principles Calculations. *Phys. Rev. Lett.* **2013**, *111*, 097401.
14. Kim, M. W. *et al*. Effect of Orbital Rotation and Mixing on the Optical Properties of Orthorhombic $RMnO_3$ (, Pr, Nd, Gd, and Tb). *Phys. Rev. Lett.* **2006**, *96*, 247205.
15. Freysoldt, C. *et al*. First-principles calculations for point defects in solids. *Rev. Mod. Phys.* **2014**, *86*, 253.
